# Case of Sub-acute Colitis, with Suppuration

**Published:** 1871-07

**Authors:** James S. Whitmire

**Affiliations:** Metamora, Ill.


### Article V.—*Case of Sub-acute Colitis, with Suppuration.*

By James S. Whitmire, M.D., Metamora, Ill.

A. E. J., aged 24, called at our office March 1, 1871. He is now employed in a dry goods store in a neighboring town; but previous to his entering this establishment, he had been clerking for several years in a retail grocery store in this place. He is now complaining of a steady aching pain in the hypogastrium, extending to the left flexure of the colon. He complained of great aggravation of the pain on steady pressure with the hand over this region, and more or less borborygmi were induced by manipulation over this region.

His stools were very much too frequent and thin, but not specially liquid, excepting just at the close of defecation; and a great quantity of thick tenacious mucus was present in every evacuation, and very often this mucus was streaked with blood, no great amount, however, being passed at any one time. He said that he had been thus affected for about three or four months, and had, by this time, though under the care of a physician, become very much emaciated and anemic, having, in this time, lost thirty lbs. in weight.

Finding himself continually losing rather than otherwise, he removed his quarters to a friend in this vicinity for rest and treatment. We diagnosed sub-acute inflammation and ulceration of the left and, probably, the whole transverse colon. Prognosis:—recovery, if complete, will be very tedious.

We prescribed at the first a dose of castor oil to carry off the contents of the bowels, to be followed with a powder composed of bismuth sub-nit. gr. xv., pulv. ipecac. gr. j , to be taken every four hours during the daytime; also a mixture composed of

℞

| | |
|---|---|
| Spt. Nit., - - - - - - - - - | ℥ss. |
| Carbolic Acid, - - - - - - - - | gr. xv. |
| Ol. Terebinth., - - - - - - - | ʒjss. |
| Glycerine, - - - - - - - - - | ℥jss. |
| Mucil. Acaciæ, - - - - - | } āā ℥j. |
| Elixir Paregoric, - - - - - | |
| | M. |

Of this mixture he was to take one teaspoonful, in water, between each powder; the directions being that he must take the powders five times and the mixture four times per day. We also ordered six drops of the oxy-sulph. of iron, with sulphate of quinine dissolved in it, three times per day, in water, at regular meals. This iron mixture is simply a solution of sulphate of iron in nitric acid and water. A strict regimen was enjoined, without which, I assured him, all treatment or medication would be useless; hence, beef soup, with rice boiled with it, was to be his exclusive diet till otherwise ordered; none of the rice, however, was permitted to be taken, excepting so much as naturally strained through a cloth. On this treatment and regimen I kept him for eight or ten days, when we found that his stools were becoming more consistent and less frequent. We now made the same prescriptions, and continued the regimen, excepting that we allowed a medium-sized roast potato at dinner and a soft-boiled egg at breakfast. The beef soup was permitted at any time during the day when he was hungry or required drink. In about twenty days from the commencement of treatment we added gr. iij. of exsiccated sulph. alumin. to the powders, and continued the same course in regimen, etc., lessening the number of the powders to four per day, and consequently the mixture to three doses. This course was pursued for, probably, twenty days longer, when our patient was discharged cured and able to attend to his usual occupation.

Externally, along the course of the tenderness, from the commencement of the treatment, we used pustulation by means of ol. tiglii one part, and ol. terebinth. three parts, so as to keep the skin quite sore.

After the fæces began to assume some consistency, we often gave a laxative to mildly clear out the alimentary tract; for this purpose we preferred a decoction of rhei, zingiberis and bicarb. sodæ. We perceived, from time to time, as the case progressed, that the thick, tenacious mucus grew less and less; often, however, this mucus would completely envelop a lump of fæcal matter unconnected with the rest, which evidently had been formed in one of the pouches of the colon, and in that place had received its coating.

One of the points in this case is, that this young man had been tortured and racked with pain, and become so emaciated as to incapacitate him for business; yet his case was successfully treated without the least particle of opium or its preparations, save the little that was in his mixture in the form of paregoric; showing clearly, to my mind, that the carbolic acid and bismuth, used in the prescription, had a decided anæsthetic effect upon the mucous membranes with which they came in contact, independent of any other curative agency that they may have exercised in the process of cure. The excess of nitric acid in the iron mixture, probably yielded a favorable influence in the speedy and final termination of the case. This young man, no doubt, had contracted the disease from the habit of eating sugar, crackers, cheese, etc., between meals during the years that he stood in the grocery store; thus keeping the stomach constantly at work so as to produce debility of that viscus, and, hence, imperfect digestion for a long time; consequently, the food taken, instead of being properly assimilated and taken into the system for necessary repairs, became a source of irritation, and occasioned the state of things we have described; and more especially the sugar, taken at all times, producing a tendency to acidity in an enfeebled stomach, instead of being assimilated and converted into fat, either to be deposited in the tissues as a healthy organism, or taken into the circulation to keep up the normal animal temperature.

